# Characterization and potential strategies for the valorisation of the Southwest Atlantic butterfish (*Stromateus brasiliensis)*

**DOI:** 10.1007/s13197-020-04332-6

**Published:** 2020-04-23

**Authors:** Graciela Ramilo-Fernández, Carmen G. Sotelo

**Affiliations:** Instituto de Investigaciones Marinas – Consejo Superior de Investigaciones Científicas (CSIC), Eduardo Cabello 6, 36208 Vigo, Spain

**Keywords:** Valorisation, Sustainability, Discards, Polyunsaturated fatty acids, Collagen extraction, Fish protein hydrolysates

## Abstract

The waste of fish resources constitutes a serious environmental problem that must be avoided. The valorisation of by-catch species and decreasing the discard rate constitute a more efficient and sustainable use of these marine biomasses. In this work, we characterize and propose different potential uses for *Stromateus brasiliensis*, another frequently discarded (≥ 90%) and poorly studied by-catch species captured in the South Atlantic Ocean (FAO 41) by trawler fishing fleets. Furthermore, in the case of this species, freezing and frozen storage of the whole fish is the only strategy currently employed for its exploitation. The results revealed that muscle from *S. brasiliensis* presented a high content of polyunsaturated fatty acids (20.34%) and that the concentrations of both total diacyl glyceryl ethers (2.41%) and heavy metals (Hg 0.038, Pb 0.006 and Cd 0.018 mg/kg) were below the established limits for safe human consumption. Likewise, the protein hydrolysates proved to be a good source of amino acids for human consumption or animal feeding. Minced muscle blocks could be made by a mechanical separation process of the flesh, and the composition of minced muscle did not differ much from that of the whole fish. Furthermore, this process allows the incorporation of cryoprotectants and antioxidants to extend the frozen shelf life of this fatty fish. An extraction process from mechanically mixed skin and bones yielded a good source of collagen that should not be neglected.

**Electronic supplementary material:**

The online version of this article (10.1007/s13197-020-04332-6) contains supplementary material, which is available to authorized users.

## Introduction

Increases in fish consumption and the demand for protein are rising due to population growth and changes in food habits. Consumers perceive fish as a healthy food. Fish are an excellent source of proteins, as well as healthy fats, vitamins and minerals, and are therefore considered “nature’s superfood” (FAO 2017; Egerton et al. 2017). Fish resources are not inexhaustible, and the current exploitation rates are unsustainable. Fish discarding practices, in which a part of the catch is returned dead or alive to the sea for different reasons, are a global problem for the sustainability of fisheries. Additionally, incidental capture worsens the problem. The last estimation of the discard rate in the world represents less than 10% of total annual catches, lower than previous estimations. (FAO 2016). The utilization of low-value or underutilized fishes and by-products is a way of increasing the supply of fish for food and other purposes without increasing the environmental impact.

Southwest Atlantic butterfish, *Stromateus brasiliensis* (*S. brasiliensis*), is a demersal-pelagic fish that is captured as by-catch in some areas of FAO 41, where many trawlers operate targeting different species, such as the Argentinian squid (*Illex argentines*) or hake (*Merluccius hubbsi*). This butterfish species is discarded by the bottom trawler fleet with discard rates of 90% or higher (J.L. del Río., personal communication). *Stromateus brasiliensis* has been poorly studied, and its valorisation uses have not been suggested until now.

This work addresses possible uses for discarded *S. brasiliensis* based on the use of muscle, skin, and bone to obtain minced muscle blocks, protein hydrolysates, and collagen, which could then be used for several industrial applications, such as food, aquafeed, pharmaceutical and cosmetics.

*Stromateus brasiliensis* is a fatty fish (16% fat), so one problem for its conservation is lipid oxidation. Mechanical separation of the edible part and the production of minced muscle could be an alternative valorisation strategy for this underutilized resource instead of marketing the whole fish because the former method permits the addition of stabilizing agents, such as antioxidants. Additionally, we studied the utilization of fish muscle to produce protein hydrolysates. Through controlled enzymatic hydrolysis, it is possible to obtain valuable small peptides that are a source of good-quality protein that can be used in aqua feed and animal feed (Martínez-Alvarez et al. 2015) or to increase protein content in products for human consumption, such as cheese sticks and biscuits (Egerton et al. 2018). *Sardina pilchardus*, *Trachurus mediterraneus*, *Pagellus acarne*, *Boops boops*, *Scyliorhinus canicula, Micromesistius poutassou* and *Capros aper* are some of the species previously employed to obtain fish protein hydrolysates that have good nutritional composition, amino acid profile, and antioxidant activities (Chalamaiah et al. 2012; Blanco et al. 2015; Pérez-Gálvez et al. 2015).

Marine collagen is a structural protein that is present in mammalian skin, bones and connective tissue and also in skin, bones, fins, and scales from fish. Marine collagen has unique features with respect to the mammalian equivalent but has not been thoroughly valorised. Recently, collagen has been obtained and characterized from the skin and bones of some discarded fishes, such as *Chimaera monstrosa*, *Etmopterus* spp., *Galeus* spp., *S. canicula*, *Leucoraja naevus* and *Nezumia aequalis* (Sotelo et al. 2016), so the value of *S. brasiliensis* collagen is studied in this work.

## Materials and methods

### Samples

Southwest Atlantic butterfish (*S. brasiliensis*) specimens were caught (January–March 2017) in the Southwest Atlantic (FAO Area 41) by a commercial fishing vessel, frozen on board and kept frozen at − 18 °C (up to 5 months) until analysis. The average weight and length of the fish were 354.28 g and 24.85 cm, respectively.

The specimens were thawed overnight at 8 °C, and then different body parts were separated manually and mechanically for yield determination. The muscle, skin and bones were separately stored in plastic bags at − 18 °C for subsequent enzymatic hydrolysis and collagen extraction experiments. Additionally, the muscle samples and prepared minced muscle blocks were employed for the determination of biochemical composition.

### Preparation of minced muscle blocks

Headed and gutted thawed fish (3 kg) were processed in a deboning machine (Grupo Josmar, Spain). The minced muscle obtained was washed with cold water (7 °C) in a proportion of 1:3 fish/water. After 10 min, the minced muscle was dewatered in a hydraulic press. Tocopherol (225 mg/kg of minced muscle), sorbitol (4%) and sunflower oil (10 ml/kg minced muscle) were added to the minced muscle and homogenized to obtain the blocks. The minced muscle blocks were then stored in plastic bags and frozen at − 18 °C until analysis.

### Determination of biochemical composition

The proximate composition of the muscle from 10 individuals and 2 minced muscle blocks was performed. Moisture was determined by the loss of weight after heating the sample at 105 °C for 24 h. Similarly, ash content was assessed by the loss of weight after heating the dried samples in a muffle furnace at 550 °C for 24 h. Crude protein was analysed by the Kjeldahl method in a DigiPREP 500 fully automatic steam distillation system (SCP Science, Baie-D’Urfe, QC, Canada) and a TitroLine Easy Unit (Metrohm AG, Ionenstrasse, Switzerland). The conversion factor used for calculating the protein content from Kjeldahl nitrogen data was 6.25. Lipid content was determined by the Bligh and Dyer extraction method (Bligh and Dyer 1959) and gravimetric quantification of extracted lipids. The analyses of fatty acids from five lipid extracts were carried out by gas chromatography of fatty acid methyl esters (FAMEs) according to ISO standard methods (ISO 12966-2:2011 and ISO 12966-4:2015). Diacyl glyceryl ethers (DAGEs) were determined in the extracted lipids by gas chromatography–mass spectrometry (GC–MS) with a previous separation by thin-layer chromatography (TLC) (Takada et al. 1979). Inductively coupled plasma-mass spectrometry (Thermo Elemental - X7 Quadrupole ICP-MS, Thermo Scientific) was used for the determination of heavy metals (mercury, cadmium and lead). Five muscle samples were previously subjected to acid digestion using nitric acid and hydrogen peroxide according to UNE-EN 15763.

### Collagen extraction

A mixture of skin and bones from *S. brasiliensis* was thawed and homogenized using a mincer (Thermomix; Vorwerk, Germany) for 1–2 min. The resulting material was mixed with 0.1 N NaOH (1:10 w/v) for 24 h with continuous stirring at 4 °C to remove non-collagenous proteins. Solid materials were separated from the alkali solution by filtering through a 500 µm filter. Afterwards, the remaining material was washed with cold distilled water until reaching neutrality; water was removed by filtering as above, and the residue collected. This residue was thoroughly mixed with 0.5 N acetic acid (1:10 w/v) and kept at 4 °C for 24 h with continuous stirring. The acid soluble collagen (ASC) present was separated from the insoluble material by filtration (residue 1) and was precipitated by the addition of NaCl until a final concentration of 2 M was achieved. The precipitated collagen was then collected by filtration. The precipitated ASC obtained was dissolved in 0.5 N acetic acid and dialyzed with water (1 week); the dialyzed ASC (fraction 1: F1) was lyophilized using a lyophilizer (VirTis BenchTop Pro). Residue 1 was suspended again in 0.5 N acetic acid (1:10 w/v) with 0.1% pepsin and kept at 4 °C for 24 h with continuous stirring. The resulting pepsin solubilised collagen PSC (fraction 2: F2) was separated from the insoluble material (residue 2) by filtration and precipitated with NaCl, solubilised with acetic acid, and dialyzed and lyophilized as described above for ASC. Residue 2 was washed with distilled water and then stirred continuously for 48 h at 4 °C with a 0.5 M EDTA solution at pH 7.4 (1:10 w/v) for demineralising the insoluble bones; the EDTA solution was changed every 8 h and finally filtered. After this treatment, the residue was thoroughly mixed with 0.5 N acetic acid (1:10 w/v) and kept at 4 °C for 24 h with continuous stirring for extracting the remaining acid soluble collagen from the bones. The solubilised collagen obtained was salted out by the addition of NaCl until a final concentration of 2.6 M was reached; this collagen was solubilised with acetic acid, dialyzed and lyophilized as described above for ASC, obtaining fraction 3 (F3). Three specimens were used for three independent full collagen extractions.

#### Yield and characterization

The yield of collagen extracted was calculated as a percentage of the lyophilized collagen obtained from skin and bone biomass on a dry and wet basis.

SDS–polyacrylamide gel electrophoresis for the characterization of collagen fractions was performed according to Sotelo et al. (2016) with some modifications. Lyophilized collagen was dissolved in sample buffer (0.5 M Tris-HCl at pH 6.8, 10% glycerol, 2% SDS, 0.6% DTT, 0.026% bromophenol blue) at a concentration of 1 mg/ml (w/v) and heated at 100 °C for 4 min. A total of 8 µl of the treated sample was loaded per well in a 7% acrylamide—0.24% bis-acrylamide separating gel (100 × 750 × 0.75 mm) and subjected to electrophoresis at a constant current of 15 mA using a Mini-Protean II Cell system (Bio-Rad Laboratories, Hercules, CA, USA). Then, the gels were stained with 0.04% Coomassie Blue in 25% v/v ethanol and 8% v/v acetic acid for 30 min at 60 °C. Excess stain was removed with several washes of destaining solvent (25% v/v ethanol, 8% v/v acetic acid). The molecular weight of the collagen fractions was established using molecular weight standards from AMRESCO (Protein MW Marker, Wide Range): myosin (212 kDa), β-galactosidase (116 kDa), phosphorylase B (97.4 kDa), bovine serum albumin (66.2 kDa), ovalbumin (45 kDa), carbonic anhydrase (31.0 kDa), and soybean trypsin inhibitor (21.5 kDa).

#### Amino acid profile

The amino acid content in collagen was determined according to Sotelo et al. (2016). The samples were hydrolysed at 110 °C for 24 h in 6 N HCl containing 0.1% w/v phenol. HCl was vaporized, and the residues were resuspended in 20 to 50 µl of 0.2 M sodium citrate buffer at pH 2.2. Norleucine was used as an internal standard. The samples were analysed using an automated amino acid analyser (Biochrom 30 series Amino Acid Analyzer, Cambridge, UK).

### Protein hydrolysates

#### Kinetics and yield

The hydrolysis reactions were performed using the pH-stat method in a 100 ml glass reactor connected to a heated circulating bath (PolyScience, USA). The pH and temperature were controlled with a glass-combined electrode connected to a Metrohm Titrando 902 system (Metrohm, Switzerland) operated by a PC (software Tiamo 2.3). Alcalase (Alcalase Novozymes A/S, Spain) was used for hydrolysis, and triplicates for each sample were analysed according to Blanco et al. (2015). Portions of 15 g of thawed mince muscle were suspended in 75 ml of water (1:5 w/v). The pH of the mixture was adjusted to pH 8 by the addition of 1 M NaOH at 55 °C, and then 150 µl of Alcalase (1% v/w of muscle) was added to start the hydrolysis. The pH was held constant during the reaction by the continuous addition of 1 M NaOH. After 4 h of hydrolysis under constant stirring, the temperature was gradually raised to 90 °C for 5 min to inactivate the Alcalase. The hydrolysates were centrifuged at 10000 × g for 10 min at 10 °C, and the supernatants were filtered by Whatman paper (100 mm Ø). Then, the samples were stored at − 20 °C until lyophilization and further analysis. The hydrolysis degree was determined by the pH-stat method defined by Adler-Nissen (1984). The average yield of the fish protein hydrolysates was calculated by determining the weight of lyophilized hydrolysate as a percentage of the total dry weight of muscle used.

#### Amino acid profile

The amino acid profile was determined in three protein hydrolysates as described in the previous “Amino acid profile” section.

## Results and discussion

Valorisation strategies may include several options to maximize the opportunities to produce benefits of otherwise low value or discarded species. In this work, after characterizing the species, we propose several valorisation strategies.

### Characterization of *Stromateus brasiliensis*

#### Body composition

The average total length and weight of the *S. brasiliensis* specimens employed in this study were 25 cm and 354 g, respectively, similar to the ones reported in Eder and Lewis (2005). *Stromateus brasiliensis* is a fish with a small head and fins, so the yield of the edible portion represents 55% of the total fish weight when manually separated. This value is similar to other commercial fish, with fish flesh percentages of approximately 40–60 (Blanco et al. 2015). From this result, it is expected that direct human consumption of *S. brasiliensis* is feasible. The head, viscera, skin, and bones represented 7.8%, 10.4%, 16.0, and 5.7% of the total body weight, respectively. The mechanical separation yield was slightly lower (19.5%) for skin and bones and higher for muscle (66.5%) than those of the specimens obtained manually. Mechanical separation using pressure is more effective at removing the flesh adhered to the skin and central bone. There were no visible bones in the minced muscle obtained from mechanical separation; therefore, the ash value obtained was lower than that in muscle obtained from manual separation (Table 1). In both cases, low ash values (< 2%) were obtained, which indicate a good separation process (Secci et al. 2017).

**Table 1:** Proximate composition (g/100 g muscle) of *Stromateus brasiliensis* muscle and minced muscle block

	Muscle	Minced muscle block
Moisture	65.28 ± 2.12	66.34 ± 0.02
Ash	1.37 ± 0.13	0.47 ± 0.06
Protein	16.58 ± 0.99	14.61 ± 0.49
Lipid	16.08 ± 2.68	12.58 ± 1.68

The results are expressed as the average percentage of wet weight ± standard deviation

#### Biochemical composition

The biochemical composition of the muscle and minced muscle blocks is shown in Table 1. Although the moisture, ash, and lipid content obtained in this study were in accordance with those previously reported (Eder and Lewis 2005), we found a slightly higher protein content in our samples. In addition, based on its lipid content (> 5%), *S. brasiliensis* can be considered a fatty fish, similar to other pelagic fishes.

Table 2 shows the fatty acid composition of the lipids extracted from *S. brasiliensis* muscle. The fatty acid profile was in the following abundance order: oleic acid (C 18:1), palmitic acid (C 16:0), docosahexaenoic acid (C 22:6; DHA), myristic acid (C 14:0), and eicosapentaenoic acid (C 20:5; EPA). The contents of the saturated (SFAs), monounsaturated (MUFAs) and polyunsaturated (PUFAs) fatty acids were 21.04%, 28.54%, and 20.34%, respectively. In this study, we found similar values of DHA (10.92%) and EPA (5.06%), referred to as % of extracted lipids, as those reported in Stromateidei oil (8.5% and 2.5%, respectively) (Endo et al. 2005). Fish have essential fatty acids, PUFAs, which cannot be produced in the human body and must be incorporated into the diet. The consumption of such marine-derived PUFAs has been associated with the prevention of cardiovascular, metabolic and inflammatory diseases and has other effects on human health (Calder 2015). The high content of PUFAs obtained should be noted, despite the frozen storage time, during which lipid oxidation could have occurred, consequently decreasing the PUFA content.

**Table 2:** Fatty acid profiles of total lipids from *Stromateus brasiliensis* muscle

Fatty acid	%
Myristic acid (14:0)	7.21 ± 0.53
Myristoleic acid (14:1)	0.28 ± 0.03
Pentadecanoic acid (15:0)	0.39 ± 0.03
Palmitic acid (16:0)	14.71 ± 0.25
Palmitoleic acid (16:1)	4.71 ± 0.89
Heptadecanoic acid (17:0)	0.18 ± 0.04
Heptadecanoleic acid (17:1)	0.46 ± 0.09
Stearic acid (18:0)	3.81 ± 0.47
Olei acid (18:1)	27.87 ± 2.43
Linoleic acid (18:2)	0.91 ± 0.54
Linolenic acid (18:3)	0.85 ± 0.11
Stearidonic acid (18:4)	3.19 ± 0.60
Arachidic acid (20:0)	0.21 ± 0.05
Eicosenoic acid (20:1)	2.60 ± 0.88
Arachidonic acid (20:4 *n*6)	1.85 ± 0.96
Eicosatetraenoic acid (20:4 *n*3)	0.82 ± 0.38
Eicosapentaenoic acid (20:5 *n*3)	5.06 ± 0.73
Heneicosapentaenoic acid (21:5 *n*3)	0.74 ± 0.11
Docosapentaenoic acid (22:5 *n*6)	0.22 ± 0.05
Docosapentaenoic acid (22:5 *n*3)	0.87 ± 0.06
Docosahexaenoic acid (22:6)	10.92 ± 1.68
Other	12.15 ± 1.18

Values are expressed as normalized % of fatty acids (mean ± standard deviation) of the total lipids analysed

There is not a consensus about whether butterfish of the genus *Stromateus* can be safely consumed by humans because the type of fat present in these fish may produce some mild gastrointestinal issues. According to previous reports, the muscle of *Stromateidae* fish contains DAGEs, a lipid component that has been related to poor digestion and absorption processes and therefore causes mild issues (Takada et al. 1979; Lee et al. 2001; Sato et al. 2002a, b; Endo et al. 2005). For this reason, the presence of DAGEs in the lipid fraction of *S. brasiliensis* was investigated. Table 3 shows the DAGE content and composition in the muscle of the samples of *S. brasiliensis*. The total DAGE content in the lipid fraction was 2.41% but was 0.39% when referred to the muscle content. However, there are only a few studies on DAGE toxicity. Sato et al. (2002a, b) reported toxic levels of DAGEs tested in mice; these levels were equivalent to the intake of 1.5 kg DAGE for a man of 60 kg of body weight. Therefore, the content of DAGEs determined in these samples of *S. brasiliensis* does not represent a risk to human health.

**Table 3:** DAGE content and composition in *Stromateus brasiliensis* muscle

	g DAGE/100 g lipid
DAGE C14:0	0.23 ± 0.03
DAGE C15:0	0.04 ± 0.01
DAGE C16:0	0.02 ± 0.01
DAGE C16:1	1.61 ± 0.23
DAGE C17:0	0.03 ± 0.01
DAGE C18:1	0.35 ± 0.07
DAGE C18:0	0.11 ± 0.03
Total DAGEs	2.41 ± 0.37

Values represent the mean ± standard deviation of three lipid extracts

Similar purgative properties have also been described for escolar (*Lepidocybium flavobrunneum* and *Ruvettus pretiosus*) and other species; in this case, the causative compounds were the high wax ester levels in the flesh of these species (Nichols et al. 2001). However, in the previously mentioned work, the presence of wax in *Stromateidae* was excluded as a cause of diarrhoea in children after consumption of *Stromateus stellatus* (Sato et al. 2002a, b). For this reason, wax content was not determined in these samples of *S. brasiliensis*.

The concentration of heavy metals in the muscle of *S. brasiliensis* was determined. The results show a content of 0.038 ± 0.033 mg/kg for Hg, 0.006 ± 0.007 mg/kg for Pb and 0.018 ± 0.017 mg/kg for Cd. These values were all below the established limits (Hg < 0.5 mg/kg, Pb < 0.30 mg/kg, and Cd < 0.05) recommended by the Codex Alimentarius (Codex Alimentarius. International Food Standards 2015) and European regulation (EC) No 1881/2006 of 19 December 2006.

### Valorisation potential

#### Human consumption

The success of the mechanical separation process in the fish sector for the production of minced fish over the last few years had an impact on the increase of the production and consumption of this type of seafood product (Palmeira et al. 2016). Considering the small size of *S. brasiliensis* and the yield of flesh, the mechanical recovery of muscle, lipid removal and posterior stabilisation constituted a potential alternative valorisation strategy for the commercialization of the whole *S. brasiliensis*. In addition, preservation of untreated frozen fatty fishes is critical because lipids are rich in polyunsaturated acids, which are prone to oxidation (Palmeira et al. 2016). Mechanical separation of *S. brasiliensis* showed a flesh recovery of 66.5%; the posterior treatment (washing and excess water removal by a manual press) rendered 50.6% of minced muscle mainly due to the elimination of the lipid fraction.

Data on the biochemical composition of the minced muscle blocks are shown in Table 1. The samples showed a higher moisture, as expected, and a slightly lower content of proteins than those observed for *S. brasiliensis* muscle. These results are the consequence of the washing process during the minced elaboration. In the case of lipids, the contents are similar; although the original lipids are removed during the washing steps, the stabilization of the minced muscle includes the addition of a tocopherol solution of sunflower oil, which may have contributed to the recovery of lipid content. The total DAGEs content in the minced muscle blocks was 0.35%.

These results indicate that this species is appropriate for valorisation as minced muscle and constitutes a valuable intermediary for the elaboration of restructured products, such as ready-to-cook or ready-to-eat seafood (Secci et al. 2017). In addition, the inclusion of a washing step and the addition of tocopherol inhibits lipid oxidation, thus expanding the shelf life of products derived from *S. brasiliensis*.

#### Collagen extraction: yield and characterization

The process of extracting collagen from the mixture of bones and skins included three acid extractions, with one including pepsin. The yields obtained for each collagen fraction expressed as g of lyophilised collagen/100 g of skin and bones, both on a dry and wet weight basis, are shown in Online Resource 1. Yields for F1 (ASC) and F2 (PSC) were similar, between 2.3 ± 0.39 and 2.0 ± 0.29% on a wet weight basis, respectively, while the yield of F3 (ASC from bones) was considerably lower (0.2 ± 0.09%).

Similar low yields (1.5%, on a wet weight basis) were reported for the acid soluble collagen (ASC) from the skin of *Brama australis*, also a fish from the Perciformes order (Sionkowska et al. 2015).

The content of collagen extracted from frozen starry triggerfish (*Abalistes stellatus*), a fish belonging to Tetraodontiformes, was 7.1% and 12.6% for ASC and PSC, respectively (on a wet weight basis); in this case, the storage time of the fish was less than 2 months (Ahmad et al. 2016). Freezing and frozen storage may have an impact on the collagen yield. The yields of ASC and PSC from frozen shark skin (*Chiloscyllium punctatum*) were in the range of 9.38% and 8.86%, respectively, based on wet weight (Kittiphattanabawon et al. 2010). These contents for other sharks are slightly higher (11–14% for ASC on a wet weight basis, calculated from the hydroxyproline content (Sotelo et al. 2016).

Collagen extraction yields depend on different factors, such as fish species, age, season, biological conditions, nutritional status, the nature and concentration of acid or alkali used during pre-treatment, the temperature and duration of the pre-treatment, the ratio of raw material to acid solution, acid concentration, incubation temperature and time in enzymatic digestion (Li et al. 2013). In fact, biochemical changes such as increase in cross-linking between collagen fibres might lead to a decrease in collagen solubility in a variety of solvents. In addition, as mentioned before, freezing and frozen storage provoke the denaturation and aggregation of collagen fibres, in turn decreasing collagen extraction (Blanco et al. 2017).

**Fig. 1:**
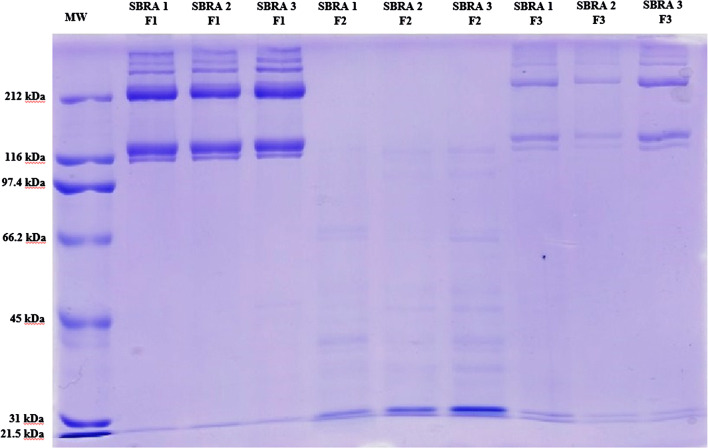
SDS–PAGE pattern of skin and bone collagen from three *Stromateus brasiliensis* individuals (SBRA 1, SBRA 2 and SBRA 3). F1, F2, and F3 are the collagen fractions extracted. MW is the molecular weight marker

The electrophoretic analysis of the three collagen fractions is shown in Fig. 1. F1 and F2 present a similar electrophoretic pattern, showing two α-chains (1 and 2) of approximately > 116 kDa, a β component of > 212 kDa, and in the case of F1 (ASC), a γ component of > 300 kDa; this lattermost component is not detected in F2 or F3. F2 is the PSC extraction; in this case, bands lower than 100 kDA are observed, showing an important susceptibility of *S. brasiliensis* skin and bone collagen to the pepsin treatment (Li et al. 2013; Ahmad and Benjakul 2010). The electrophoretic pattern of *S. brasiliensis* collagen corresponds with type I collagen, as previously reported for the skin of other fish species (Ahmad and Benjakul 2010; Wang et al. 2017).

The amino acid composition of the three fractions of collagen is shown in Table 4 as residues/1000 total amino acids. Glycine was the most abundant amino acid and represented one-third of the total amino acid residues in fractions 1 and 2, as is usually found for type I collagen. Alanine and proline were next in order of abundance, and methionine, tyrosine, histidine, and cysteine were the least abundant amino acids, as previously reported for collagens from other fishes (Kittiphattanabawon et al. 2010; Huang et al. 2011). ANOVA was performed for eight amino acid characteristics in collagen (Gly, Ala, Pro, Leu, Tyr, Asp, Glu, and HPro) to test for differences between the fractions obtained. Glycine, proline, alanine, leucine and aspartic acid showed significant differences (*P* < 0.05) between F1 and F3, and alanine, proline and tyrosine also showed difference between F2 and F3. No significant differences were observed in glutamic acid and hydroxyproline between the three fractions. These imino acids (proline and hydroxyproline) have been related to the structural integrity of collagen (Huang et al. 2011). Their contents in the three extracted collagen fractions of *S. brasiliensis* (F1: 165; F2:162; F3:144) were lower than those of the skin collagen of bamboo shark (ASC: 204/1000 residues and PSC: 207/1000 residues) (Kittiphattanabawon et al. 2010) but similar to those of the skin of brown backed toadfish (170/1000 residues) (Senaratne et al. 2006). The degree of hydroxylation of proline also plays a role in the stability of the helix structure of collagen. F1, F2 and F3 of *S. brasiliensis* collagen had hydroxylation degrees of 39%, 41% and 43%, respectively, similar to those of bigeye snapper skin collagen (40% in skin and 41% in bones) (Kittiphattanabawon et al. 2005) and unicorn leatherjacket skin collagen (40%) (Ahmad and Benjakul 2010). Finally, the degree of hydroxylation of lysine, which influences the establishment and stabilization of crosslinks, was calculated for F1, F2, and F3, and the results were not different among the fractions (22%, 21%, and 20%).

**Table 4:** Amino acid profile (residues/1000 total amino acid residues) of ASC (acid soluble collagen) and PSC (pepsin soluble collagen) from *Stromateus brasiliensis* skin and bones (mean ± standard deviation)

Amino acid	Fraction 1	Fraction 2	Fraction 3
Aspartic acid	49.12 ± 1.05^a^	54.09 ± 1.69^ab^	60.72 ± 7.04^b^
Threonine	26.65 ± 1.33	28.97 ± 1.73	32.22 ± 4.08
Serine	54.08 ± 0.39	55.57 ± 1.40	57.21 ± 2.04
Glutamic acid	71.30 ± 0.98^ns^	77.26 ± 1.10^ns^	83.60 ± 9.80^ns^
Hydroxyproline	65.12 ± 1.61^ns^	66.31 ± 2.17^ns^	62.68 ± 18.57^ns^
Proline	100.30 ± 2.91^a^	95.62 ± 7.09^ab^	81.68 ± 5.65^c^
Glycine	324.53 ± 6.34^a^	305.59 ± 3.59^ab^	267.80 ± 31.16^b^
Alanine	122.19 ± 2.13^a^	120.57 ± 6.27^ab^	107.10 ± 2.70^c^
Cysteine	2.16 ± 0.42	2.53 ± 0.51	3.87 ± 0.70
Valine	22.03 ± 1.52	23.32 ± 2.60	33.36 ± 5.37
Methionine	16.07 ± 0.05	14.36 ± 0.44	23.00 ± 2.06
Isoleucine	10.86 ± 1.41	12.76 ± 2.50	19.70 ± 5.18
Leucine	25.53 ± 1.08^a^	29.94 ± 3.09^ab^	40.46 ± 8.16^b^
Tyrosine	3.95 ± 0.97^ab^	3.83 ± 0.57^a^	8.38 ± 2.93^b^
Phenylalanine	13.86 ± 0.75	15.49 ± 0.57	17.91 ± 3.01
Hydroxylysine	7.61 ± 1.26	8.10 ± 1.39	8.90 ± 1.53
Histidine	5.45 ± 0.14	5.3 ± 0.43	8.85 ± 1.78
Lysine	27.62 ± 0.76	29.66 ± 1.23	34.68 ± 7.81
Arginine	51.59 ± 1.14	47.60 ± 0.16	47.88 ± 0.83
Imino acids (Pro + HPro)	165	162	144
Pro hydroxylation (%)	39	41	43
Lys hydroxylation (%)	22	21	20

Different letters indicate significant differences among means for the same amino acid (*P* < 0.05); *ns* indicates no significant differences

#### Protein hydrolysates: kinetics, yield and amino acid profile

The hydrolysis curve from *S. brasiliensis* mince muscle with Alcalase can be seen in Online Resource 2. After an initial rapid phase (1 h), the rate of enzymatic hydrolysis decreased, reaching a steady-state phase after 180 min of hydrolysis. The hydrolysis degree achieved within 240 min was 26.49 ± 0.49%. These results were similar to those previously described for boardfish muscle (*C. aper*) with Alcalase (Blanco et al. 2015); lower hydrolysis degrees were obtained for blue whiting (*M. poutassou*) muscle (20%) minced with Alcalase at 180 min (Egerton et al. 2018).

The protein hydrolysate yield for *S. brasiliensis* with Alcalase was 49.92 ± 1.33% and is expressed as the amount of dry hydrolysate from 100 g weight of minced muscle used. Different yield values have been reported in a variety of fish species, mainly underutilized, from various by-products, with different enzymes and hydrolysis conditions (Kristinsson and Rasco 2000).

Table 5 shows the amino acid composition and content of the hydrolysates, expressed as residues/1000 total amino acids. Glutamic acid (144/1000), aspartic acid (107/1000) and alanine (93/1000) were the most abundant amino acids present in the hydrolysates. These results agree with those previously reported for the hydrolysis of boardfish (*C. aper*) muscle with Alcalase (Blanco et al. 2015). Glutamic and aspartic acid were also the main amino acids in Bluewing searobin (*Prionotus punctatus*) hydrolysates with Alcalase (dos Santos et al. 2011).

**Table 5:** Amino acid profile (residues/1000 total amino acid residues) of *Stromateus brasiliensis* hydrolysates prepared with Alcalase (mean ± standard deviation)

Amino acid	Hydrolysates
Aspartic acid	107.28 ± 0.43
Threonine	55.01 ± 0.84
Serine	58.06 ± 1.61
Glutamic acid	144.01 ± 0.64
Hydroxyproline	3.25 ± 0.55
Proline	42.01 ± 0.61
Glycine	84.24 ± 2.28
Alanine	92.53 ± 0.75
Cysteine	6.21 ± 0.54
Valine	46.68 ± 0.97
Methionine	31.21 ± 1.13
Isoleucine	37.88 ± 0.31
Leucine	83.37 ± 0.45
Tyrosine	26.98 ± 0.27
Phenylalanine	31.52 ± 0.41
Hydroxylysine	1.90 ± 2.40
Histidine	19.19 ± 0.26
Lysine	81.19 ± 0.85
Arginine	44.25 ± 0.79

The amino acid composition of proteins and hydrolysates is important to determine their nutritional value, and the quantities of nine essential amino acids (EAA) are often evaluated to assess this value for human and animal foods and feeds (Gehring et al. 2011). In addition, amino acid composition influences the functional properties of protein hydrolysates (dos Santos et al. 2011).

Thr, Leu, Met, Lys, Val, Ile, Trp, His, and Phe are considered EAA for humans. Five of the ten most abundant amino acids in *S. brasiliensis* hydrolysates (Leu, Lys, Thr, Val, and Arg) are among these EAAs. Lys and Met are two amino acids that have been traditionally used to assure the adequate composition of amino acids in fish meal. In the case of the *S. brasiliensis* protein hydrolysates, lysine was found to be abundant (81/1000 residues), indicating the potential utilization of *S. brasiliensis* for animal feed applications.

## Conclusion

The present work demonstrates that undervalued fish species can be valorised using complementary processes, such as producing blocks of minced muscle, hydrolysing fish protein together with extracting collagen/gelatine to valorise the rest of the raw materials (see Online Resource 3). The capture of some fish species from distant waters can be improved if the products are adequately processed and preserved, such as in the case of the production of minced muscle blocks, in which cryoproctectans and antioxidants are included. We have shown that *S. brasiliensis* is an interesting source of amino acids, polyunsaturated fatty acids and collagen. The results from minced muscle block composition and yields suggest that *S. brasiliensis* would be suitable for the elaboration of restructured products, such as hamburgers or fish balls, for human consumption.

## Electronic supplementary material

Below is the link to the electronic supplementary material.

Supplementary material 1 (PDF 238 kb)

## References

[CR1] Adler-Nissen J (1984). Control of the proteolytic reaction and of the level of bitterness in protein hydrolysis processes. J Chem Technol Biotechnol Biotechnol.

[CR2] Ahmad M, Benjakul S (2010). Extraction and characterisation of pepsin-solubilised collagen from the skin of unicorn leatherjacket (*Aluterus monocerous*). Food Chem.

[CR3] Ahmad M, Nirmal NP, Chuprom J (2016). Molecular characteristics of collagen extracted from the starry triggerfish skin and its potential in the development of biodegradable packaging film. RSC Adv.

[CR4] Blanco M, Sotelo CG, Pérez-Martín RI (2015). Hydrolysis as a valorization strategy for unused marine food biomass: boarfish and small-spotted catshark discards and by-products. J Food Biochem.

[CR5] Blanco M, Vázquez J, Pérez-Martín R, Sotelo C (2017). Hydrolysates of fish skin collagen: an opportunity for valorizing fish industry byproducts. Mar Drugs.

[CR9] Bligh EG, Dyer WJ (1959). A rapid method of total lipid extraction and purification. Canadian J Biochem Physiol.

[CR6] Calder PC (2015). Functional roles of fatty acids and their effects on human health. J Parenter Enter Nutr.

[CR7] Chalamaiah M, Dinesh Kumar B, Hemalatha R, Jyothirmayi T (2012). Fish protein hydrolysates: proximate composition, amino acid composition, antioxidant activities and applications: a review. Food Chem.

[CR8] dos Santos SDA, Martins VG, Salas-Mellado M, Prentice C (2011). Evaluation of functional properties in protein hydrolysates from bluewing searobin (*Prionotus punctatus*) obtained with different microbial enzymes. Food Bioprocess Technol.

[CR10] Eder EB, Lewis MN (2005). Proximate composition and energetic value of demersal and pelagic prey species from the Southwestern Atlantic Ocean. Mar Ecol Prog Ser.

[CR11] Egerton S, Culloty S, Whooley J, Stanton C, Paul Ross R (2017). Boarfish (*Capros aper*): review of a new capture fishery and its valorization potential. ICES J Mar Sci.

[CR12] Egerton S, Culloty S, Whooley J, Stanton C, Ross RP (2018). Characterization of protein hydrolysates from blue whiting (*Micromesistius poutassou*) and their application in beverage fortification. Food Chem.

[CR13] Endo Y, Tagiri-Endo M, Kimura K (2005). Rapid determination of iodine value and saponification value of fish oils by near-infrared spectroscopy. J Food Sci.

[CR14] FAO (2016) The state of world fisheries and aquaculture 2016. In: Contributing to food security and nutrition for all. Rome, p 200. http://www.fao.org/3/a-i5555e.pdf. Accessed 23 Aug 2018

[CR15] FAO (2017) FAO working for SDG14: healthy oceans for food security, nutrition and resilient communities, pp 10–11. http://www.fao.org/3/a-i7298e.pdf. Accessed 9 Dec 2018

[CR16] Gehring CK, Gigliotti JC, Moritz JS, Tou JC, Jaczynski J (2011). Functional and nutritional characteristics of proteins and lipids recovered by isoelectric processing of fish by-products and low-value fish: a review. Food Chem.

[CR17] Huang YR, Shiau CY, Chen HH, Huang BC (2011). Isolation and characterization of acid and pepsin-solubilized collagens from the skin of balloon fish (*Diodon holocanthus*). Food Hydrocoll.

[CR18] Kittiphattanabawon P, Benjakul S, Visessanguan W, Nagai T, Tanaka M (2005). Characterisation of acid-soluble collagen from skin and bone of bigeye snapper (*Priacanthus tayenus*). Food Chem.

[CR19] Kittiphattanabawon P, Benjakul S, Visessanguan W, Kishimura H, Shahidi F (2010). Isolation and characterisation of collagen from the skin of brownbanded bamboo shark (*Chiloscyllium punctatum*). Food Chem.

[CR20] Kristinsson HG, Rasco BA (2000). Fish protein hydrolysates: production, biochemical, and functional properties. Crit Rev Food Sci Nutr.

[CR21] Lee JS, Kim JH, Lee TS, Park JH (2001). Diacyl glyceryl ethers as the causative agent in the diarrheal episode associated with consumption of *Stromateus stellatus*. Korean J Fish Aquat Sci.

[CR22] Li ZR, Wang B, Chi CF, Zhang QH, Gong YD, Tang JJ, Luo HY, Ding GF (2013). Isolation and characterization of acid soluble collagens and pepsin soluble collagens from the skin and bone of Spanish mackerel (*Scomberomorous niphonius*). Food Hydrocoll.

[CR23] Martínez-Alvarez O, Chamorro S, Brenes A (2015). Protein hydrolysates from animal processing by-products as a source of bioactive molecules with interest in animal feeding: a review. Food Res Int.

[CR25] Nichols PD, Mooney BD, Elliott NG (2001). Unusually high levels of non-saponifiable lipids in the fishes escolar and rudderfish: identification by gas and thin-layer chromatography. J Chromatogr A.

[CR26] Palmeira KR, Mársico ET, Monteiro MLG, Lemos M, Conte CA (2016). Ready-to-eat products elaborated with mechanically separated fish meat from waste processing: challenges and chemical quality. CYTA J Food.

[CR27] Pérez-Gálvez R, García-Moreno PJ, Morales-Medina R, Guadix A, Guadix EM (2015). Bile acid binding capacity of fish protein hydrolysates from discard species of the West Mediterranean Sea. Food Funct.

[CR28] Sato T, Seo HS, Endo Y, Fujimoto K (2002). Diacyl glyceryl ether as the major muscle lipid in *Stromateus stellatus* and its hydrolyzability by lipase and oral acute toxicity on mice. Bull Jpn Soc Sci Fish.

[CR29] Sato T, Seo H-S, Endo Y, Fujimoto K (2002). Diacyl glyceryl ether as the major muscle lipid in *Stromateus stellatus* and its hydrolyzability by lipase and oral acute toxicity on mice. Nippon Suisan Gakkaishi.

[CR30] Secci G, Borgogno M, Mancini S, Paci G, Parisi G (2017). Mechanical separation process for the value enhancement of atlantic horse mackerel (*Trachurus trachurus*), a discard fish. Innov Food Sci Emerg Technol.

[CR31] Senaratne LS, Park PJ, Kim SK (2006). Isolation and characterization of collagen from brown backed toadfish (*Lagocephalus gloveri*) skin. Bioresour Technol.

[CR32] Sionkowska A, Kozłowska J, Skorupska M, Michalska M (2015). Isolation and characterization of collagen from the skin of *Brama australis*. Int J Biol Macromol.

[CR33] Sotelo, CG., Comesaña, MB, Ariza, PR, Pérez-Martín, RI (2016) Characterization of collagen from different discarded fish species of the west coast of the Iberian Peninsula. J Aquat Food Prod Technol 25(3):388–399. 10.1080/10498850.2013.865283

[CR34] Takada K, Kamiya H, Hashimoto Y (1979). Studies on lipids of some stromateidei fishes. Bull Jpn Soc Sci Fish.

[CR35] Wang S, Sun X, Zhou D (2017). Physicochemical characteristics and fibril-forming properties of collagen from paddlefish (*Polyodon spathula*) and Globefish (*Fugu flavidus*) skin byproducts. Food Sci Technol.

