# Supplementary material for: Characterization and potential strategies for the valorisation of the Southwest Atlantic butterfish (Stromateus brasiliensis)
Source: J Food Sci Technol. 2020 Apr 23;57(8):2994–3003. doi: 10.1007/s13197-020-04332-6 (PMC7316936; doi:10.1007/s13197-020-04332-6)

Characterization and potential strategies for the valorisation of the Southwest Atlantic butterfish (*Stromateus brasiliensis)*

Online Resource 1: Yields (%) of ASC and PSC from skin and bones.

	**Yield (%)**	
**Samples**	**Dry weight basis**	**Wet weight basis**
**Fraction 1**	5.0 ± 0.83	2.3 ± 0.39
**Fraction 2**	4.3 ± 0.61	2.0 ± 029
**Fraction 3**	0.4 ± 0.19	0.2 ± 0.09
**Combined yield**	9.7 ± 0.1	4.5 ± 0.2

The results are the mean values of three extractions ± standard deviation

Online Resource 2: Hydrolysis curve for *Stromateus brasiliensis* with Alcalase. Values are the mean of three triplicates analysis. Error bars represent standard deviation.

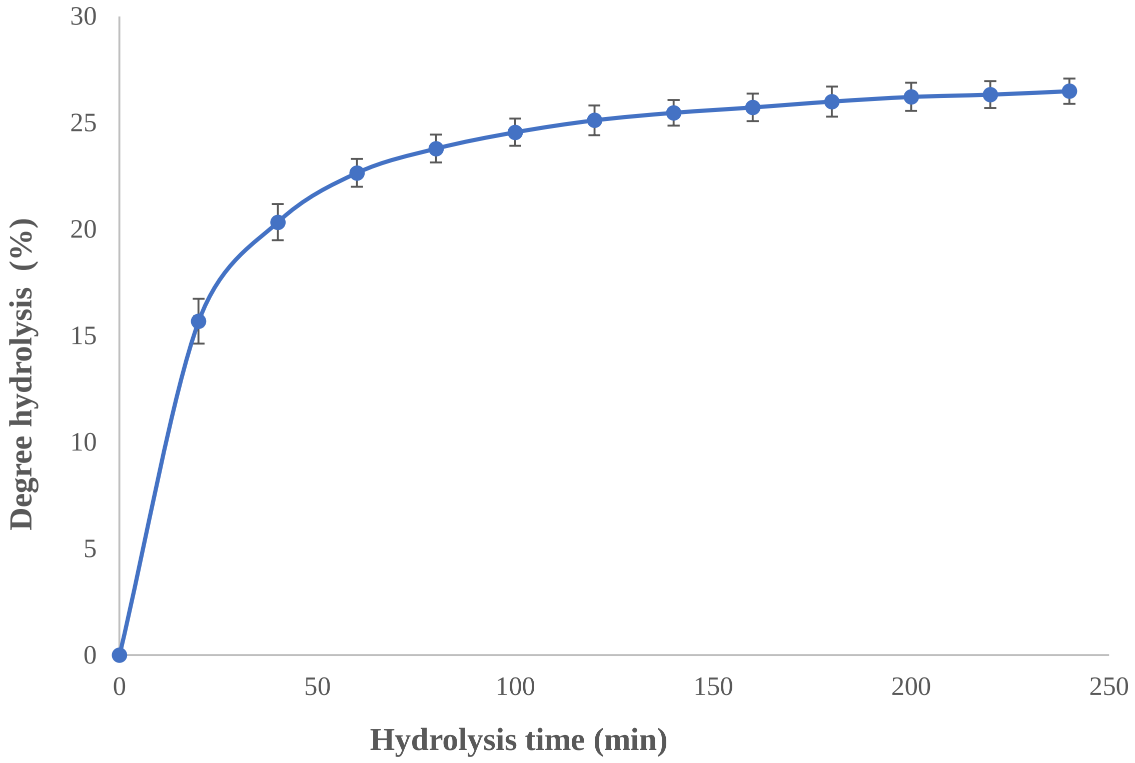

Online Resource 3: Scheme of *Stromateus brasiliensis* potential uses.

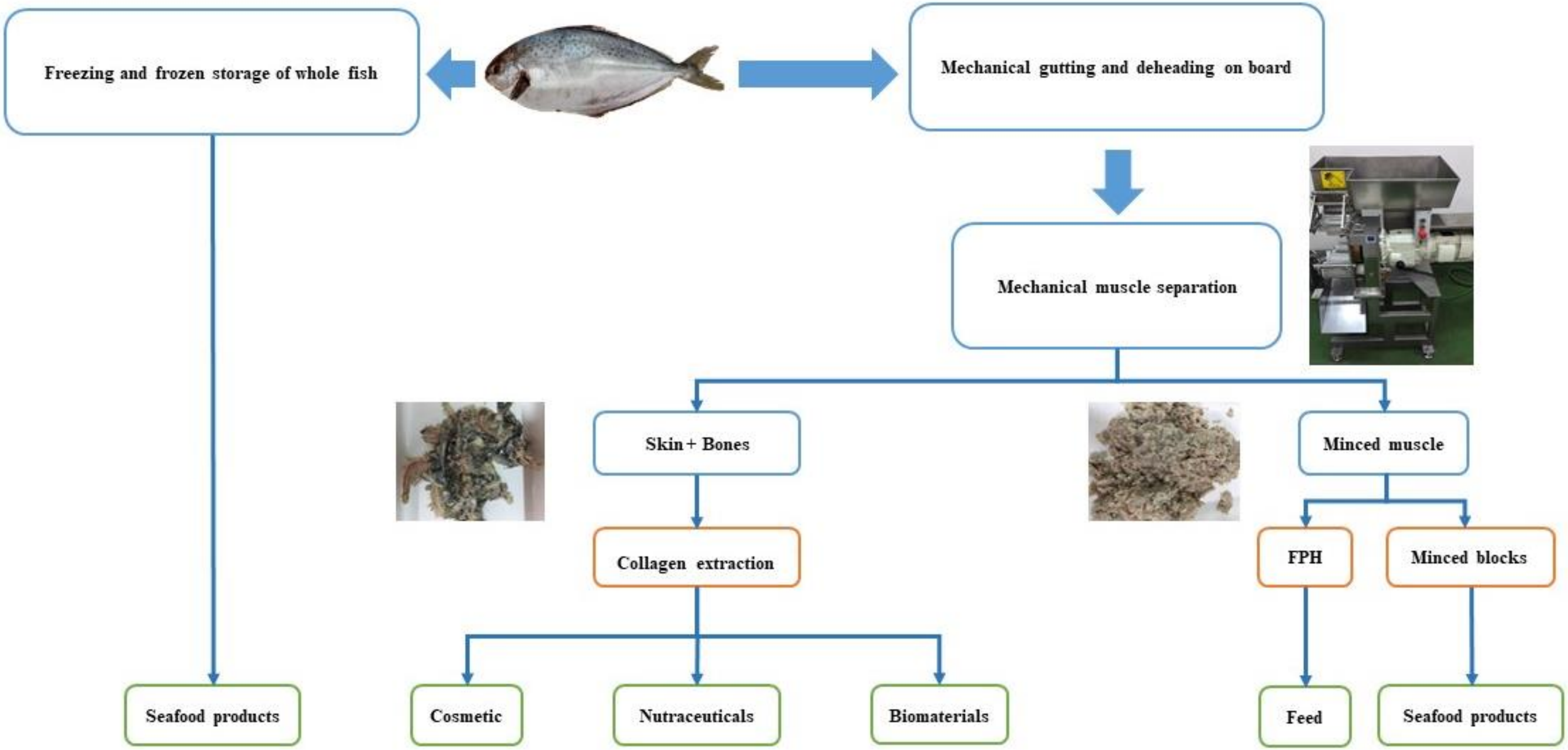

Supplement: Supplementary file 1 — Supplementary material 1 (PDF 238 kb) [file 13197_2020_4332_MOESM1_ESM.pdf]
